# Exchange protein directly activated by cAMP 2 is required for corticotropin‐releasing hormone‐mediated spine loss

**DOI:** 10.1111/ejn.14487

**Published:** 2019-07-09

**Authors:** Zhong Xie, Peter Penzes, Deepak P. Srivastava

**Affiliations:** ^1^ Department of Physiology Feinberg School of Medicine Northwestern University Chicago IL USA; ^2^ Department of Psychiatry and Behavioral Sciences Feinberg School of Medicine Northwestern University Chicago IL USA; ^3^ Center for Autism and Neurodevelopment Northwestern University Chicago IL USA; ^4^ Department of Basic and Clinical Neuroscience Maurice Wohl Clinical Neuroscience Institute Institute of Psychiatry, Psychology and Neuroscience King's College London London UK; ^5^ MRC Centre for Neurodevelopmental Disorders King's College London London UK

**Keywords:** cortical neurons, dendritic spine, excitatory synapse, rap, small GTPase, stress

## Abstract

Corticotropin‐releasing hormone is produced in response to acute and chronic stress. Previous studies have shown that activation of the corticotropin‐releasing hormone receptor 1 (CRHR1) by corticotropin‐releasing hormone results in the rapid loss of dendritic spines which correlates with cognitive dysfunction associated with stress. Exchange protein directly activated by cAMP (EPAC2), a guanine nucleotide exchange factor for the small GTPase Rap, plays a critical role in regulating dendritic spine morphology and has been linked with CRHR1 signalling. In this study, we have tested whether EPAC2 links corticotropin‐releasing hormone with dendritic spine remodelling. In primary rat cortical neurons, we show that CRHR1 is highly enriched in the dendritic spines. Furthermore, we find that EPAC2 and CRHR1 co‐localize in cortical neurons and that acute exposure to corticotropin‐releasing hormone induces spine loss. To establish whether EPAC2 was required for corticotropin‐releasing hormone–mediated spine loss, we knocked‐down EPAC2 in cortical neurons using a short hairpin RNA‐mediated approach. In the presence of *Epac2* knocked‐down, corticotropin‐releasing hormone was no longer able to induce spine loss. Taken together, our data indicate that EPAC2 is required for the rapid loss of dendritic spines induced by corticotropin‐releasing hormone and may ultimately contribute to responses to acute stress.

## Graphical abstract

Corticotropin‐releasing hormone (CRH) is produced in response to acute and chronic stress and has been shown to cause the rapid loss of dendritic spines in hippocampal neurons. In this study, we show that CRH induces a rapid loss of dendritic spine in cortical neurons. Moreover, we find that exchange protein directly activated by cAMP 2 (EPAC2), a guanine nucleotide exchange factor for the small GTPase Rap and regulator of dendritic spines, is required for CRH‐mediated spine loss.

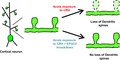

**Abbreviations**
aCSF: artificial cerebral spinal fluid
APV: amino‐phosphonovalerate
CRH: corticotropin‐releasing hormone
CRHR1: corticotropin‐releasing hormone receptor 1
DIV: days in vitro
GEF: guanine nucleotide exchange factor
GFP: green fluorescent protein
PBS: phosphate‐buffered saline

## 1 INTRODUCTION

Corticotropin‐releasing hormone (CRH) is a 41‐amino acid neuropeptide that is an important regulator of hormonal, behavioural and autonomic responses to stress (Henckens, Deussing, & Chen, 2016; Sanders & Nemeroff, 2016). CRH is expressed in discrete regions within the central nervous system (Grammatopoulos, 2012; Henckens et al., 2016), and CRH receptors are expressed in multiple brain regions (Henckens et al., 2016). The CRH receptor type 1 (CRHR1) is a 7‐transmembrane G‐protein–coupled receptor that transmits signals via the Gsα‐mediated regulation of cAMP (Grammatopoulos, 2012; Inda et al., 2016). The CRH peptide has been shown to be released locally within the amygdala, hippocampus and cortex, and to be involved in the modulation of cognition, including memory and anxiety, during stress (Henckens et al., 2016; Sanders & Nemeroff, 2016). Moreover, chronic exposure to CRH may have long‐lasting detrimental effects (Henckens et al., 2016; Maras & Baram, 2012). Interestingly, acute exposure to CRH also results in the loss of dendritic spine on CA1 hippocampal neurons; this effect could be blocked by CRHR1 antagonism (Andres et al., 2013; Chen, Dube, Rice, & Baram, 2008). CRH‐mediated spine loss in the hippocampus has been linked with RhoA signalling (Chen et al., 2013), as well as a nectin‐3/afadin complex (Wang et al., 2013).

Exchange protein directly activated by cAMP 2 (EPAC2, also known as cAMP‐GEFII or RapGEF4) is a signalling protein present in forebrain postsynaptic densities (Woolfrey et al., 2009). This protein has been shown to be involved in a range of cognitive function including social behaviours (Srivastava et al., 2012) and learning and memory (Yang et al., 2012). Interestingly, recent studies have also implicated the *Epac2* gene in the response to stress, anxiety and depression (Aesoy et al., 2018; Zhou et al., 2016). Interestingly, EPAC2 has been suggested to mediate CRH/CRHR1 coupling to the ERK‐MAPK pathway (Grammatopoulos, 2012; Inda et al., 2016). Moreover, EPAC2 has also been shown to regulate dendritic spine morphology, motility and density (Srivastava et al., 2012; Woolfrey et al., 2009). Based on these previous studies, we hypothesized that EPAC2 might play a role in CRH‐/CRHR1‐mediated spine alterations.

Here, we investigated the presence of CRHR1 at dendritic spines of primary cortical neurons. We examined whether CRHR1 and EPAC2 co‐localized within cortical neurons and whether acute exposure to CRH‐altered spine density in cortical neurons. Finally, we tested whether EPAC2 was required for CRH‐mediated spine loss.

## 2 MATERIALS AND METHODS

### 2.1 Reagents

Corticotropin‐releasing hormone was purchased from Bio‐Techne (“CRF”, Cat. No. 1151). Antibodies used: green fluorescent protein (GFP) mouse monoclonal (MAB3580; Merck; 1:1,000); EPAC2 mouse monoclonal (A‐7, Santa Cruz, 1:200); CRHR1 rabbit polyclonal (EB08035; Everest Biotech; 1:500). Control and *Epac2*‐shRNA constructs, expressing shRNA sequences and GFP, were previously described (Woolfrey et al., 2009).

### 2.2 Neuronal culture and transfections

Cortical neuronal cultures, derived from both sexes, were prepared from E18 Sprague–Dawley rat embryos as previously described (Srivastava, Woolfrey, & Penzes, 2011). Animals were used in accordance with ACUC institutional and national guidelines under approved protocols. Briefly, cells dissociated from embryonic tissue were plated onto 18‐mm glass coverslips coated with poly‐d‐lysine (0.2 mg/mL), at a density of 3 × 10^5^. Neurons were cultured in feeding media: neurobasal medium supplemented with 2% B27, 0.5 mM glutamine and 1% penicillin:streptomycin (Life Technologies). Neuronal cultures were maintained in the presence of 200 μM d,l‐amino‐phosphonovalerate (APV) beginning on day 4 in vitro (DIV 4) in order to maintain neuronal health for long‐term culturing.

Primary cortical neurons were transfected with eGFP, control (scram‐)RNAi or *Epac2*‐RNAi at DIV 21, using Lipofectamine 2000. Briefly, 2–4 μg of plasmid DNA was mixed with Lipofectamine 2000 and incubated for 4 hr, before being replaced with fresh feeding media. Transfections were allowed to proceed for 2–5 days, after which cells were used for pharmacological treatment or immunocytochemistry.

### 2.3 Pharmacological treatments of neuronal cultures

Treatments were performed in artificial cerebral spinal fluid (aCSF): (in mM) 125 NaCl, 2.5 KCL, 26.2 NaHCO_3,_ 1 NaH_2_PO_4_, 11 glucose, 5 HEPES, 2.5 CaCl_2_, 1.25 MgCl_2_ and 0.2 APV. CRH was dissolved in H_2_O (10 mM), serially diluted to 1 μM in aCSF and applied directly to neuronal cultures at a final concentration of 100 nM. Final amount of H_2_O was <0.01%; vehicle control was made up of H_2_O‐lacking compound. Treatment time was 30 min.

### 2.4 Immunocytochemistry

Neurons were washed in phosphate‐buffered saline (PBS), fixed in 4% formaldehyde/4% sucrose PBS for 10 min at room temperature, followed by incubation in methanol pre‐chilled to −20°C for 10 min at 4°C. Fixed neurons were then permeabilized and blocked simultaneously (2% normal goat serum, and 0.1% Triton X‐100 in PBS with 4% sucrose). Primary antibodies were incubated overnight: cells were washed with PBS followed by incubation with secondary antibodies for 1 hr at room temperature. Coverslips were mounted onto microslides using ProMount Gold antifade.

### 2.5 Quantitative analysis of spine morphologies and immunofluorescence

Confocal images of immuno‐stained neurons were acquired with a Zeiss LSM5 Pascal confocal microscope and a 63× objective (NA = 1.4). Two‐dimensional maximum projection images were reconstructed and analysed using MetaMorph software (Molecular Devices, Sunnyvale). Morphometric analysis was performed on spines from two dendrites (secondary or tertiary branches), totalling 100 μm, from each neuron. Co‐localiztion analysis was carried out previously described (Srivastava et al., 2011). Briefly, puncta for each protein were defined as puncta that contained immunofluorescence staining greater than background of the reciprocal protein co‐stained; background fluorescence was the average background intensity from five regions of interest plus two standard deviations. Analysis was limited to two secondary or tertiary dendrites, totalling 100 μm. Cultures directly compared were stained simultaneously and imaged with the same acquisition parameters. For each condition, 13‐18 neurons from at least 3 separate experiments were used. Analyses were performed blind to condition and on sister cultures. In the green/magenta colour scheme, co‐localization is indicated by white overlap.

### 2.6 Statistical analysis

**Figure 1:**
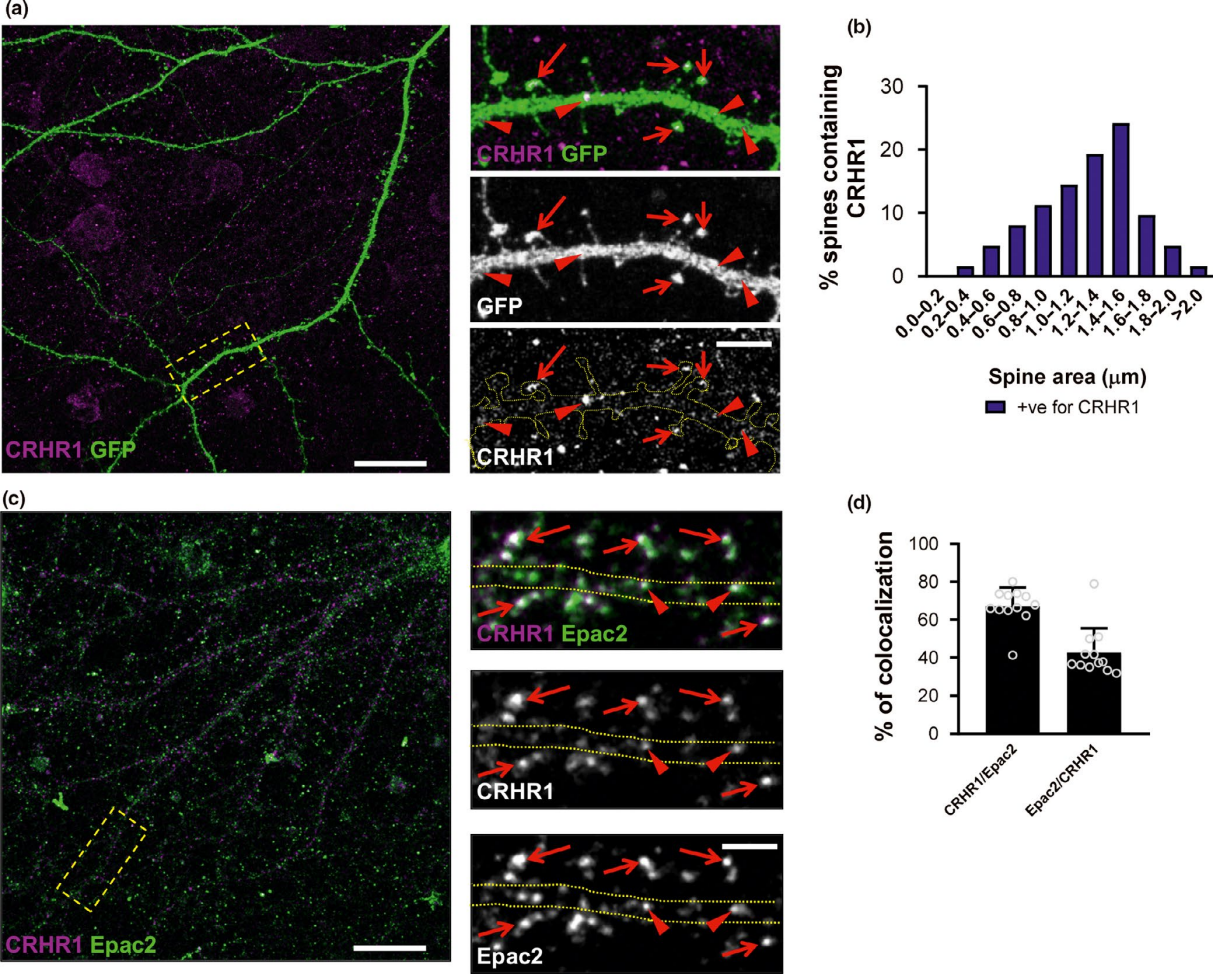
CRHR1 co‐localizes with EPAC2 in primary cortical neurons. (a) Representative confocal microscopic images of a GFP‐expressing cortical neuron double immunostained for GFP and CRHR1. The yellow box indicates the region of the dendrite displayed in magnified insets. Red arrows indicate spines enriched for CRHR1. Red arrowheads denote CRHR1 puncta within dendrites. (b) Histogram of the frequency of CRHR1 staining in spines of various sizes. The greatest enrichment of CRHR1 was observed in spines with an area of 1.0–1.6 μm. (c) Representative confocal microscopic images of a cortical neuron double immunostained for EPAC2 and CRHR1. The yellow box indicates the region of the dendrite displayed in magnified insets. Red arrows indicate spine‐like structures where overlapping CRHR1 and EPAC2 puncta were observed. Red arrowheads denote co‐localizing CRHR1 and EPAC2 puncta within dendrites. (d) Bar graph indicates quantitative measures of respective co‐localization of immunofluorescent puncta. Scale bars: 20 μm for the main panels, 5 μm for the insets. [Colour figure can be viewed at http://wileyonlinelibrary.com]

**Figure 2:**
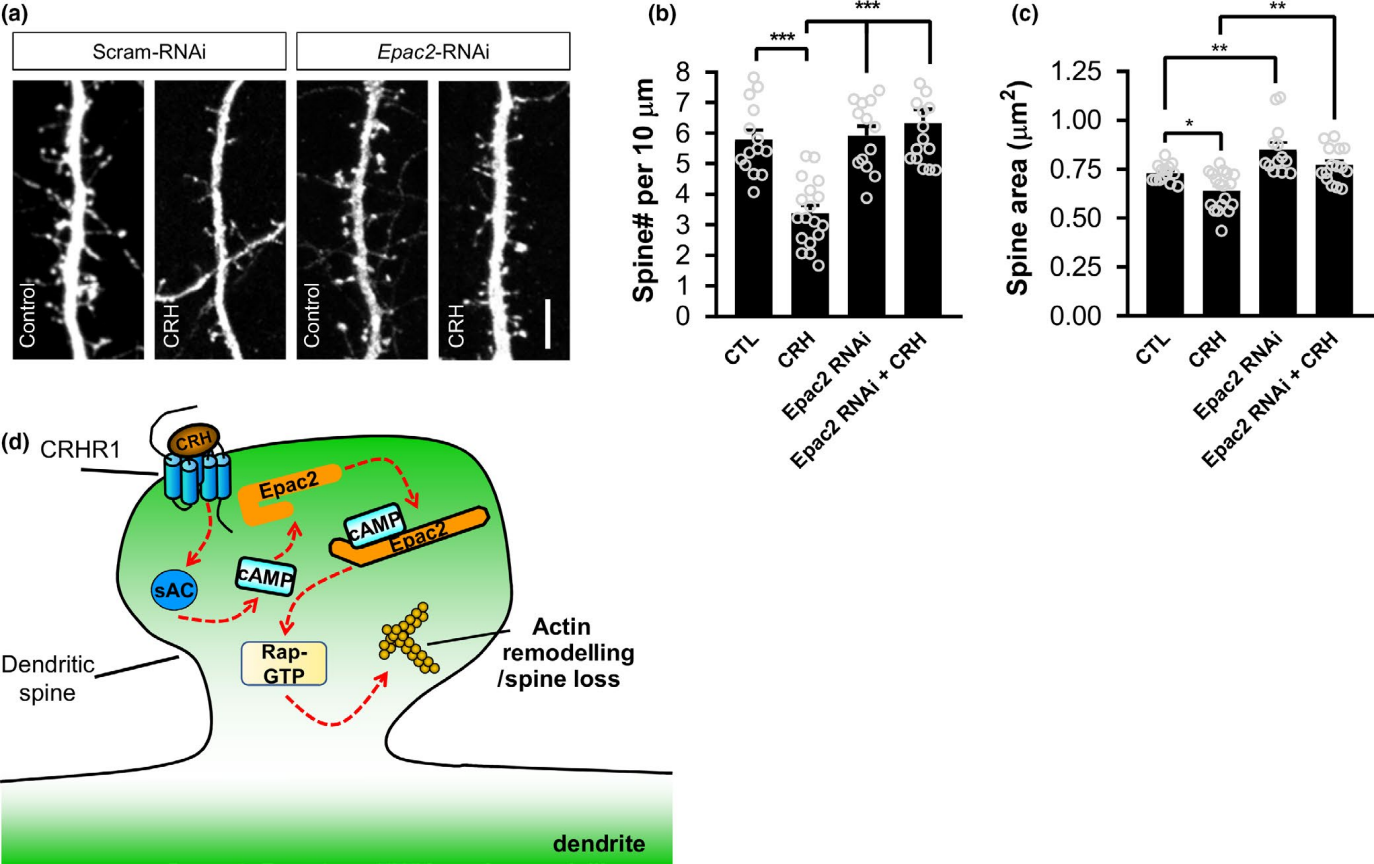
EPAC2 is required for CRH‐induced rapid spine loss in primary cortical neurons. (a) Representative confocal microscopic images of dendrites from cortical neurons expressing either a control shRNA (Scram_RNAi) or *Epac2*‐shRNA (Epac2_RNAi). Neurons were treated with either 100 nM CRH or vehicle control for 30 min. Scale bar: 5 μm. (b) Bar graph indicates quantitative measures of linear spine density. Treatment with CRH for 30 min induced a significant loss of spines (spines per 10 μm: control, 5.79 ± 0.31; CRH, 3.39 ± 0.25; *Epac2*‐RNAi + control, 5.92 ± 0.32; *Epac2*‐RNAi + CRH, 6.33 ± 0.45; *F*(3, 56) = 17.60, *P* < 0.0001; and Tukey post‐hoc, ***=*P* < 0.001). This effect was not observed in neurons in which EPAC2 was knocked‐down. (c) Quantitative measures of spine area reveal that CRH treatment results in a significant reduction of spine size; *Epac2‐*shRNA increases spine size, whereas *Epac2‐*shRNA expressing cells show no effect of CRH (spine area (μm^2^): control, 0.73 ± 0.012; CRH, 0.64 ± 0.023; *Epac2*‐shRNA + control, 0.85 ± 0.036; *Epac2*‐shRNA + CRH, 0.77 ± 0.023; *F*(3, 56) = 13.3, *P* < 0.001; and Tukey post‐hoc, *=*P* < 0.05, **=*P* < 0.01). (c) Proposed model of CRHR1/EPAC2 signalling complex and effect on actin remodelling/loss of spines. [Colour figure can be viewed at http://wileyonlinelibrary.com]

All statistical analysis was performed in GraphPad. Differences in quantitative immunofluorescence and dendritic spine number were probed by one‐way ANOVAs with Tukey correction for multiple comparisons. Error bars represent standard deviations in Figure 1d and standard errors of the mean in Figure 2b and c.

## 3 RESULTS

### 3.1 CRHR1 localizes to dendritic spines and co‐localizes with EPAC2 in cortical neurons

Previous studies have shown that CRHR1 localizes in dendritic spines of CA1 hippocampal neurons (Andres et al., 2013; Chen et al., 2010). Whilst CRHR1 expression in cortical regions has been reported, whether or not this receptor is expressed in spines of cortical neurons have yet to be established. To investigate this, we examined the localization of CRHR1 in cortical neurons (Figure 1a). Immunoreactive puncta for CRHR1 could be observed within the somatodendritic compartment of neurons. In individual neurons, CRHR1 clustered along dendrites, with prominent puncta evident near or at the base of dendritic spines (red arrowheads) and distinct clusters within spine heads (red arrows, Figure 1a). To determine whether CRHR1 localized to a specific subpopulation of dendritic spines, we classified spines containing CRHR1 according to dendritic spine area. Spines with an area of less than 1.0 μm were designated as “small”; thus forming weaker synapses, whereas spines with an area of larger than 1.0 μm were designated as “large”; making stronger synaptic connections. Of the spines that contained CRHR1, the majority (~60%) were large (Figure 1b). These data indicate that in primary cortical neurons, CRHR1 is enriched at synapses of a subset of large dendritic spines.

Pharmacological studies have suggested that CRHR1 may signal via the EPAC proteins (Grammatopoulos, 2012; Inda et al., 2016; Traver, Marien, Martin, Hirsch, & Michel, 2006). EPAC2 is the predominant EPAC protein expressed in cortical neurons with mature dendritic morphology and is highly enriched in dendritic spines (Woolfrey et al., 2009). Therefore, we reasoned that CRHR1 may co‐localize with EPAC2 in cortical neurons. Consistent with our previous work (Woolfrey et al., 2009), we observed EPAC2 was along dendrites and in spine‐like structures (Figure 1c). Consistent with our data describing the localization of CRHR1 in cortical neurons, CRHR1 was also observed along dendrites and spine‐like structures. Moreover, CRHR1 and EPAC2 were found to co‐localize along dendrites (red arrowheads) and in a subset of spine‐like structures (red arrows; Figure 1c). Quantification of co‐localization revealed that ~70% of CRHR1 puncta co‐localized with EPAC2, whereas only ~40% of EPAC2 puncta co‐localized with CRHR1 (Figure 1d). Taken together, these data indicate that CRHR1 is ideally positioned to interact with and signal via EPAC2 at synapses.

### 3.2 CRH‐mediated spine loss is dependent on EPAC2 in cortical neurons

As our data indicated that CRHR1 co‐localizes with EPAC2, we hypothesized that CRH‐mediated spine loss may be mediated by this Rap GEF. To test this, we treated primary cortical neurons expressing a control shRNA (scram‐RNAi) or an shRNA specific for *Epac2* (*Epac2*‐RNAi; Figure S1; Woolfrey et al., 2009) with CRH. Consistent with previous reports, 30 min of CRH exposure resulted in a significant loss of dendritic spines (Figure 2). *Epac2*‐RNAi alone had no effect on spine linear density; however, treatment of cortical neurons expressing *Epac2*‐RNAi with CRH no longer resulted in a reduction in dendritic spine density (Figure 2b). Examination of spine morphology revealed that CRH treatment resulted in an overall decrease in spine size; this effect was no longer evident in neurons treated with CRH and expressing *Epac2*‐RNAi (Figure 2c). Taken together, these data suggest that CRH signals via EPAC2 to induce the rapid loss of dendritic spines.

## 4 DISCUSSION

Previous studies have shown that CRH signalling via CRHR1 can cause the rapid and persistent loss of dendritic spines in hippocampal neurons (Chen et al., 2008, 2010). This loss of spine density is further correlated with memory defects associated with acute stress (Chen et al., 2010). In this study, we build upon these findings to show that in primary cortical neurons, CRHR1 localizes to synapses in dendritic spines, where it co‐localizes with the Rap GEF EPAC2. Moreover, acute exposure to CRH resulted in the rapid loss of dendritic spines, an effect that was attenuate by the knockdown of *Epac2*. Taken together, these data indicate that CRH signalling via a CRHR1/EPAC2‐dependent signalling pathway is responsible for the actions of this hormone in regulating the density of dendritic spines and contributing to acute stress effects (Figure 2d).

Stress is a biologically important event that can have both positive and negative effects on brain function. Multiple lines of evidence have demonstrated that stress can induce a range of morphological changes in neuronal and synaptic structure. CRH is released in response to stress, and recent findings have shown that blocking the CRHR1 receptor was sufficient to block stress‐induced spine loss (Chen et al., 2008, 2010). Interestingly, in hippocampal neurons, CRH‐mediated spine loss on CA1 hippocampal neurons is dependent on synaptic‐activity (Andres et al., 2013). Data indicate that EPAC2 is required for CRH‐dependent spine loss on cortical neurons. Whether CRH acts via the regulation of both EPAC2 and synaptic‐activity to induce spine loss, or whether these are independent mechanisms dependent on cell type is currently unclear.

Moreover, recent studies have shown that EPAC2 expression is increased in response to acute stress and is involved in controlling cellular responses to acute stress (Aesoy et al., 2018). Indeed, animals lacking the EPAC proteins display increased anxiety and depressive behaviours (Srivastava et al., 2012; Yang et al., 2012). Interestingly, our previous work has shown that EPAC2 is a key regulator of dendritic spine structural plasticity in response to a number of extrinsic stimuli (Srivastava et al., 2012; Woolfrey et al., 2009). Consistent with these studies, our current data indicate that EPAC2 is required for mediating CRH‐induced spine loss. In hippocampal neurons, CRH‐mediated spine loss is dependent on RhoA activity (Chen et al., 2013) as well as a nectin‐3/afadin complex (Wang et al., 2013). Afadin is a direct target of Epac‐Rap1 signalling, and a Rap1‐afadin complex has been shown to regulate RhoA activity and cytoskeletal dynamics in endothelia cells (Birukova, Tian, Tian, Higginbotham, & Birukov, 2013). Thus, an intriguing possibility is that these signalling molecules cooperate to induce spine loss following CRH‐activity. Interestingly, it has recently been shown that CRHR1 engages atypical soluble adenylate cyclase to signal to EPAC proteins (Inda et al., 2016) indicating an indirect interaction between these two proteins.

It is also interesting to note that EPAC2 has been associated with anxious and depressive behaviours as well as in stress responses (Aesoy et al., 2018; Yang et al., 2012; Zhou et al., 2016). Furthermore, EPAC2 plays an important role in cognitive function, including learning and memory (Srivastava et al., 2012; Yang et al., 2012). Given that CRH and CRHR1 have strongly been implicated in stress‐mediated effects on these cognitive functions (Henckens et al., 2016; Maras & Baram, 2012), the data presented in this study suggest that a CRH/CRHR1/EPAC2 pathway may be critical for these effects. Taken together, these data reveal a novel mechanism involving EPAC2, by which CRH‐induced rapid modulation of dendritic spines can occur. Future studies will be required to understand whether and how this pathway is involved in mediating responses to chronic stress at both the morphological and behavioural levels.

## CONFLICT OF INTEREST STATEMENT

The authors have no competing interests to declare.

## AUTHOR CONTRIBUTIONS

P.P and D.P.S. were responsible for the conception and design of the work. Z.X. and D.P.S. were responsible for data collection, analysis and interpretation. Drafting and critical revision of the article was carried out by P.P. and D.P.S. Final approval of the version to be published was confirmed by Z.X., P.P. and D.P.S.

## Data Availability

Primary data material can be accessed by contacting the corresponding authors.

## References

[ejn14487-bib-0001] Aesoy, R. , Muwonge, H. , Asrud, K. S. , Sabir, M. , Witsoe, S. L. , Bjornstad, R. , … Bakke, M. (2018). Deletion of exchange proteins directly activated by cAMP (Epac) causes defects in hippocampal signaling in female mice. PLoS ONE, 13, e0200935. 30048476 10.1371/journal.pone.0200935 PMC6062027

[ejn14487-bib-0002] Andres, A. L. , Regev, L. , Phi, L. , Seese, R. R. , Chen, Y. , Gall, C. M. , & Baram, T. Z. (2013). NMDA receptor activation and calpain contribute to disruption of dendritic spines by the stress neuropeptide CRH. Journal of Neuroscience, 33, 16945–16960. 10.1523/JNEUROSCI.1445-13.2013 24155300 PMC3807024

[ejn14487-bib-0003] Birukova, A. A. , Tian, X. , Tian, Y. , Higginbotham, K. , & Birukov, K. G. (2013). Rap‐afadin axis in control of Rho signaling and endothelial barrier recovery. Molecular Biology of the Cell, 24, 2678–2688. 10.1091/mbc.E13-02-0098 23864716 PMC3756920

[ejn14487-bib-0004] Chen, Y. , Dube, C. M. , Rice, C. J. , & Baram, T. Z. (2008). Rapid loss of dendritic spines after stress involves derangement of spine dynamics by corticotropin‐releasing hormone. Journal of Neuroscience, 28, 2903–2911. 10.1523/JNEUROSCI.0225-08.2008 18337421 PMC2409370

[ejn14487-bib-0005] Chen, Y. , Kramar, E. A. , Chen, L. Y. , Babayan, A. H. , Andres, A. L. , Gall, C. M. , … Baram, T. Z. (2013). Impairment of synaptic plasticity by the stress mediator CRH involves selective destruction of thin dendritic spines via RhoA signaling. Molecular Psychiatry, 18, 485–496. 10.1038/mp.2012.17 22411227 PMC3440527

[ejn14487-bib-0006] Chen, Y. , Rex, C. S. , Rice, C. J. , Dube, C. M. , Gall, C. M. , Lynch, G. , & Baram, T. Z. (2010). Correlated memory defects and hippocampal dendritic spine loss after acute stress involve corticotropin‐releasing hormone signaling. Proceedings of the National Academy of Sciences of the United States of America, 107, 13123–13128. 10.1073/pnas.1003825107 20615973 PMC2919915

[ejn14487-bib-0007] Grammatopoulos, D. K. (2012). Insights into mechanisms of corticotropin‐releasing hormone receptor signal transduction. British Journal of Pharmacology, 166, 85–97. 10.1111/j.1476-5381.2011.01631.x 21883143 PMC3415640

[ejn14487-bib-0008] Henckens, M. J. , Deussing, J. M. , & Chen, A. (2016). Region‐specific roles of the corticotropin‐releasing factor‐urocortin system in stress. Nature Reviews Neuroscience, 17, 636–651. 10.1038/nrn.2016.94 27586075

[ejn14487-bib-0009] Inda, C. , Dos Santos Claro, P. A. , Bonfiglio, J. J. , Senin, S. A. , Maccarrone, G. , Turck, C. W. , & Silberstein, S. (2016). Different cAMP sources are critically involved in G protein‐coupled receptor CRHR1 signaling. Journal of Cell Biology, 214, 181–195. 10.1083/jcb.201512075 27402953 PMC4949449

[ejn14487-bib-0010] Maras, P. M. , & Baram, T. Z. (2012). Sculpting the hippocampus from within: Stress, spines, and CRH. Trends in Neurosciences, 35, 315–324. 10.1016/j.tins.2012.01.005 22386641 PMC3423222

[ejn14487-bib-0011] Sanders, J. , & Nemeroff, C. (2016). The CRF system as a therapeutic target for neuropsychiatric disorders. Trends in Pharmacological Sciences, 37, 1045–1054. 10.1016/j.tips.2016.09.004 27717506 PMC5121012

[ejn14487-bib-0012] Srivastava, D. P. , Jones, K. A. , Woolfrey, K. M. , Burgdorf, J. , Russell, T. A. , Kalmbach, A. , … Penzes, P. (2012). Social, communication, and cortical structural impairments in Epac2‐deficient mice. Journal of Neuroscience, 32, 11864–11878. 10.1523/JNEUROSCI.1349-12.2012 22915127 PMC3520089

[ejn14487-bib-0013] Srivastava, D. P. , Woolfrey, K. M. , & Penzes, P. (2011). Analysis of dendritic spine morphology in cultured CNS neurons. Journal of Visualized Experiments, e2794. 21775964 10.3791/2794 PMC3196192

[ejn14487-bib-0014] Traver, S. , Marien, M. , Martin, E. , Hirsch, E. C. , & Michel, P. P. (2006). The phenotypic differentiation of locus ceruleus noradrenergic neurons mediated by brain‐derived neurotrophic factor is enhanced by corticotropin releasing factor through the activation of a cAMP‐dependent signaling pathway. Molecular Pharmacology, 70, 30–40. 10.1124/mol.106.022715 16569708

[ejn14487-bib-0015] Wang, X. D. , Su, Y. A. , Wagner, K. V. , Avrabos, C. , Scharf, S. H. , Hartmann, J. , … Schmidt, M. V. (2013). Nectin‐3 links CRHR1 signaling to stress‐induced memory deficits and spine loss. Nature Neuroscience, 16, 706–713. 10.1038/nn.3395 23644483

[ejn14487-bib-0016] Woolfrey, K. M. , Srivastava, D. P. , Photowala, H. , Yamashita, M. , Barbolina, M. V. , Cahill, M. E. , … Penzes, P. (2009). Epac2 induces synapse remodeling and depression and its disease‐associated forms alter spines. Nature Neuroscience, 12, 1275–1284. 10.1038/nn.2386 19734897 PMC2754861

[ejn14487-bib-0017] Yang, Y. , Shu, X. , Liu, D. , Shang, Y. , Wu, Y. , Pei, L. , … Lu, Y. (2012). EPAC null mutation impairs learning and social interactions via aberrant regulation of miR‐124 and Zif268 translation. Neuron, 73, 774–788. 10.1016/j.neuron.2012.02.003 22365550 PMC3307595

[ejn14487-bib-0018] Zhou, L. , Ma, S. L. , Yeung, P. K. , Wong, Y. H. , Tsim, K. W. , So, K. F. , … Chung, S. K. (2016). Anxiety and depression with neurogenesis defects in exchange protein directly activated by cAMP 2‐deficient mice are ameliorated by a selective serotonin reuptake inhibitor, Prozac. Translational Psychiatry, 6, e881 10.1038/tp.2016.129 27598965 PMC5048194

