# Supplementary material for: Exchange protein directly activated by cAMP 2 is required for corticotropin‐releasing hormone‐mediated spine loss
Source: Eur J Neurosci. 2019 Jul 9;50(7):3108–14. doi: 10.1111/ejn.14487 (PMC6821562; doi:10.1111/ejn.14487)
Supplement: Supplementary file 1 [file EJN-50-3108-s001.pdf]

Exchange protein directly activated by cAMP 2 is required for corticotropin-releasing hormone-mediated spine loss

Zhong Xie[1], Peter Penzes[1,2,3]*, Deepak P. Srivastava[1,4,5]*

[1]Department of Physiology, Feinberg School of Medicine, Northwestern University, Chicago, IL, USA; [2]Department of Psychiatry and Behavioral Sciences, Feinberg School of Medicine, Northwestern University, Chicago, IL, USA; [3]Center for Autism and Neurodevelopment, Northwestern University, Chicago, USA, IL; [4]Department of Basic and Clinical Neuroscience, Maurice Wohl Clinical Neuroscience Institute, Institute of Psychiatry, Psychology and Neuroscience, King's College London, London, SE5 9RT, UK; [5]MRC Centre for Neurodevelopmental Disorders, King's College London, London SE1 1UL, UK.

*Correspondence:

Peter Penzes, Department of Physiology, Feinberg School of Medicine, Northwestern University, Chicago, IL, USA, e-mail: p-penzes@northwestern.edu; or Deepak Srivastava, Department of Basic and Clinical Neuroscience, King's College London, London, SE5 9RT, UK, e-mail: deepak.srivastava@kcl.ac.uk

Running title: EPAC2 mediates CRH-induced spine loss

Supplemental Information

Supplemental Figures

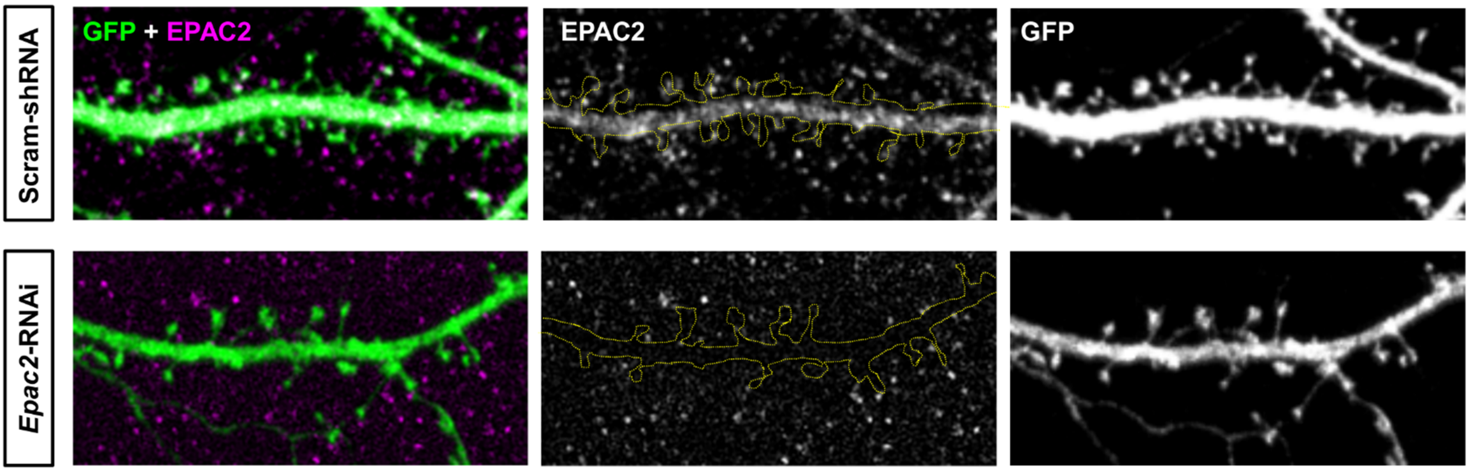

Supplemental Figure 1: EPAC2 knockdown by shRNA. Representative confocal images of cortical neurons expressing Scram-RNAi (control shRNA) or *Epac2*-RNAi (*Epac2*-shRNA) and double immunostained for GFP (to outline cell morphology) and EPAC2. Yellow dotted lines outline cell morphology, as determined by GFP staining, in EPAC2 gray scale images. In presence of *Epac2*-RNAi, endogenous EPAC2 is reduced.
